# Promising Targets for Cancer Immunotherapy: TLRs, RLRs, and STING-Mediated Innate Immune Pathways

**DOI:** 10.3390/ijms18020404

**Published:** 2017-02-14

**Authors:** Kai Li, Shuai Qu, Xi Chen, Qiong Wu, Ming Shi

**Affiliations:** School of Life Science and Technology, Harbin Institute of Technology, Harbin 150080, China; likai.19870816@163.com (K.L.); qs1996neptune@163.com (S.Q.); www.candychen@foxmail.com (X.C.); kigo@hit.edu.cn (Q.W.)

**Keywords:** cancer immunotherapy, innate immunity, Toll-like Receptors, RIG-I-like Receptors, Stimulator of Interferon Genes

## Abstract

Malignant cancers employ diverse and intricate immune evasion strategies, which lead to inadequately effective responses of many clinical cancer therapies. However, emerging data suggest that activation of the tolerant innate immune system in cancer patients is able, at least partially, to counteract tumor-induced immunosuppression, which indicates triggering of the innate immune response as a novel immunotherapeutic strategy may result in improved therapeutic outcomes for cancer patients. The promising innate immune targets include Toll-like Receptors (TLRs), RIG-I-like Receptors (RLRs), and Stimulator of Interferon Genes (STING). This review discusses the antitumor properties of TLRs, RLRs, and STING-mediated innate immune pathways, as well as the promising innate immune targets for potential application in cancer immunotherapy.

## 1. Introduction

The innate immune system is an evolutionarily ancient and dominant defense mechanism against pathogen infection [1]. The innate immune system recognizes pathogen-associated molecular patterns (PAMPs) through pattern recognition receptors (PRRs) to initiate immune response [2]. These PRRs can also recognize some endogenous damage-associated molecular patterns (DAMPs), including various tumor-derived antigens [3]. Thus, activation of the innate immune system might play a role in recognizing and counteracting malignant cells, in which immune surveillance holds the key physiologic function [4]. Despite the fact that evidence has shown that the resistance mechanisms within cancer microenvironment might orchestrate tumor evasion from immune surveillance, it is clear that activation of the tolerant innate immune system can effectively, at least partially, suppress tumor proliferation and metastasis [5,6]. In the cancer microenvironment, the presence or absence of appropriate innate immune responses may be the key determinant of immunotherapy success.

Defined innate immune mechanisms involving cancer immunotherapy include, but are not limited to antitumor immune responses elicited by recognition of tumor-derived antigens by Toll-like receptors (TLRs), as well as sensation of tumor-derived DNA by the stimulator of interferon gene (STING) and retinoic acid-inducible gene-I (RIG-I)-like Receptors (RLRs) [7,8,9]. The crucial regulators of these innate antitumor immune mechanisms have attracted increasing attention as therapeutic targets. A deeper knowledge of these therapeutically relevant targets in TLRs, RLRs, and STING-mediated innate immune pathways is leading toward novel immunotherapeutic strategies for cancer treatment.

Tremendous advances in cancer immunotherapeutic strategies have been witnessed in recent years. Targeting the innate immune pathway has been considered as an effective auxiliary strategy to prevent tumor escape, and the enhancement of the sensitivity of innate immune effectors to tumor-derived antigens could facilitate evading tumor evasion [10]. Meanwhile, the immunotherapeutic strategies that simultaneously target the innate and adaptive immune system are effective in the eradication of large tumors [11,12]. Therefore, further understanding and exploration of the mechanisms of innate immune is expected to culminate in the development of effective cancer immunotherapies.

## 2. Targeting Toll-Like Receptors in Cancer Immunotherapy

### 2.1. Toll-Like Receptors

**Figure 1:**
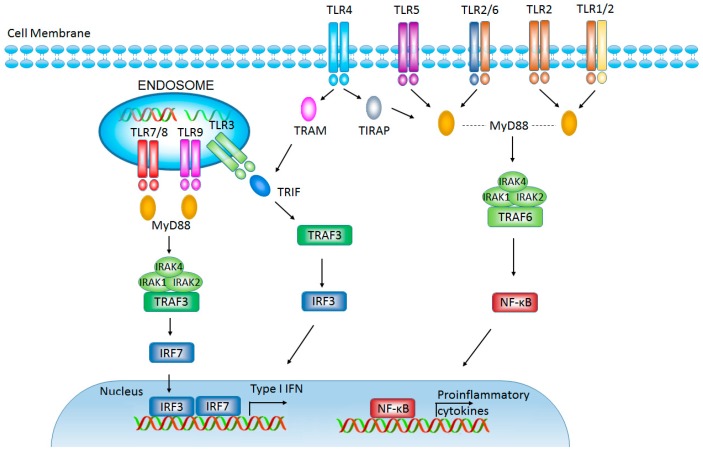
Toll-like receptors (TLRs) and TLR-mediated signaling pathway. TLR3, TLR4, TLR5, TLR7, and TLR9 form homodimers to deliver their signal after interacting with their specific ligands. TLR1 and TLR6 recognize their ligands as heterodimers with TLR2. TLR3, TLR7/8, and TLR9 are intracellular TLRs and are involved in the recognition of nucleic acids. TLRs, except for TLR3, deliver a signal through myeloid differentiation protein 88 (MyD88) pathway to activate nuclear factor-κB (NF-κB). TLR3 and TLR4 can signal through TIR domain-containing adaptor inducing IFN-β (TRIF) pathway to activate type I interferon (IFN) response. IRF: interferon regulatory factors; TRAF: tumor necrosis factor receptor-associated factor; IRAK: interleukin-1 receptor-associated kinase; TRAM: TRIF-related adaptor molecule; TIRAP: Toll-IL-1R domain-containing adapter protein.

TLRs are mammalian homologues of drosophila Toll protein, which are regarded as critical PRRs in innate immunity. There are 10 TLRs (TLR1-10) that have been identified in human. TLR1, TLR2, TLR4, TLR5, and TLR6 are expressed on the cell surface, while TLR3, TLR7, TLR8, and TLR9 are found on the membrane of endosomes. Each conserved TLR contains transmembrane domain, ectodomain (ligand binding domain with leucine-rich repeats motif), and cytosolic Toll/IL-1 receptor (TIR) domain [13]. The ectodomain of TLRs recognizes PAMPs of invading pathogens and initiates TLR activation in early innate immune response. The initial TLR activation triggers the association of receptor TIR domain with TIR domain of adapter proteins (myeloid differentiation protein 88 (MyD88)/ Toll-IL-1R domain-containing adapter protein (TIRAP) and TIR domain-containing adaptor inducing IFN-β (TRIF)/TRIF-related adaptor molecule (TRAM)) leading to activation of nuclear factor-κB (NF-κB) and interferon regulatory factor signaling pathways, which ultimately results in the activation of immune response to clean pathogens [13,14] (Figure 1).

TLRs exhibit specificity for ligand recognition. Different TLRs specifically recognize district PAMPs and DAMPs. TLR2 recognizes lipoproteins and peptidoglycans, TLR3 recognizes viral double-stranded RNA/viral RNA analogue polyinosinic-polycytidylic acid (poly I:C), TLR4 recognizes lipopolysaccharides (LPS), TLR5 recognizes bacterial flagellin, TLR7/8 recognizes single-stranded RNA, and TLR9 recognizes CpG-containing oligodeoxynucleotides (CpG-ODN) [13,15,16]. The endogenous matrix proteoglycan versican, and heat-shock proteins (HSPs) are recognized by TLR2/6, TLR2, and TLR4 [17,18]. Among these ligands, TLR2/4 agonist Bacillus Calmette-Guérin (BCG), TLR4 agonist monophosphoryl lipid A (MPL), and TLR7 agonist imiquimod have been approved for use in cancer therapy [19,20,21,22,23]. Meanwhile, several TLR ligands have been investigated as targeted cancer therapeutic agents under pre-clinical and clinical evaluation. Phase II study of TLR5 agonist Flagellin-derived CBLB502 (Entolimod) is currently ongoing in patients with advanced solid tumors [24]. TLR7 agonist 852A is also being investigated in melanoma patients [25]. More recently, TLR3 agonist poly (I:C)/poly-ICLC and TLR9 agonist synthetic CpG-ODN are being investigated as cancer vaccine adjuvant in pre-clinical and clinical trials [25,26,27] (Table 1).

### 2.2. TLRs Influence Tumor Evasion from Immune Surveillance

Recent studies have suggested that TLRs expression is not confined to immune cells, but also found in various tumor cells [37,38,39]. Emerging evidence has shown that TLRs expression on both tumor cells and immune cells relates to cancer progression [38,40]; however, different TLRs might display quite opposite outcomes in cancer development [39,41]. TLRs of immune cells serve as fundamental sensor molecules of the host innate immune system, which detect tumor-derived antigens and evoke primary anti-tumor immune response [42]. Activated TLRs trigger cytotoxic activity of immune effector cells that eliminates nascent tumor by direct lysis via distinct signaling pathways [43]. The timely elimination of tumor antigens by TLRs can further prevent the establishment of inflammatory tumor microenvironment [44]. In immune effector cells such as dendritic cells (DCs) and macrophages, the TLR-triggered cytotoxic activity and its ability of boosting anti-tumor immune responses make the TLRs innate immune defense against cancer.

To survive the host innate defense mechanisms, cancer cells have developed strategies to evade or counteract the signaling through the TLR pathways, which create an advantageous microenvironment for their propagation. Activation of certain TLRs on tumor cells enhances immune suppression through decreasing cytotoxicity of immune cells, increasing the production of pro-inflammatory factors, and triggering an abnormal form of tissue repair in tumor foci, and finally resulting in tumor evasion from immune surveillance [45,46,47].

The function of TLRs in immune and cancer cells seems complex, and the regulatory mechanisms on how to achieve targeted activation of TLRs on immune cells or cancer cells are still unclear. Perhaps distinct TLR signaling sub-pathways or unidentified downstream adaptor molecules are activated in cancer and/or innate immune cells during tumorigenesis; another possible explanation is that chronological order of TLR activation in immune cells or cancer cells markedly affect the subsequent activation and induced effectors [14,21]. The unsettled issue has become a hot topic that affects the potential of TLR agonists or antagonists as anti-tumor immunotherapeutic agents.

### 2.3. TLRs Facilitate Inflaming Metastasis of Cancer

**Figure 2:**
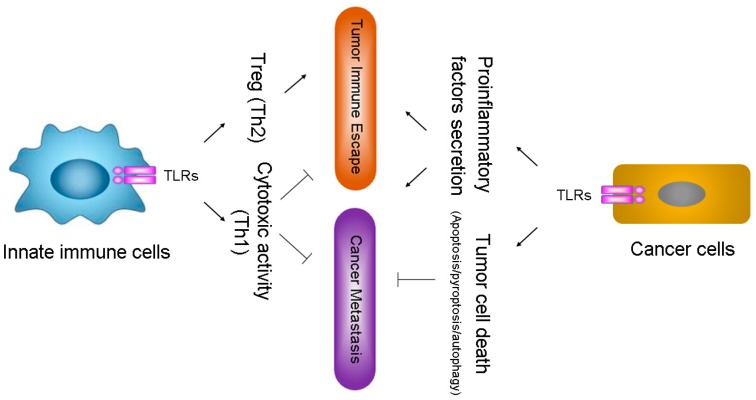
Toll-like receptors (TLRs) and cancer. TLRs activation in innate immune cells, such as dendritic cells (DCs) and macrophages, and tumor cells lead to a complex cascade reaction, which determines the role of TLRs in cancer development. The activation of TLRs in innate immune cells lead to either T cytotoxic responses (Th1) or regulatory T cell (Treg) responses (Th2). The activation of certain TLRs, such as TLR2, 4, and 9, in cancer cells promotes tumor immune escape and cancer metastasis by increasing the secretion of pro-inflammatory factors, while the activation of TLR3, 4, 5, and 7 might inhibit cancer via inducing cell death (including apoptosis, pyroptosis, and autophagic cell death) of cancer cells.

Inflaming metastasis has been proposed to be the seventh hallmark of malignant cancers [48]. Evidence has shown that chronic inflammation induced by TLRs is associated with tumorigenesis, and TLRs on cancer cells facilitate induction of a pro-inflammatory tumor microenvironment, which further supports tumor invasion and metastasis [49,50]. Additionally, the elevated level of a number of TLRs has also been linked to promoting cancer cell survival, proliferation, and metastasis in multiple types of cancer [50,51] (Figure 2).

### 2.4. Triggering of TLRs Induces Strong Immune Response against Multiple Cancers

The strategies of promoting innate immune responses by TLRs against cancer do not require complex and patient-specific therapeutic agents; meanwhile, the increasingly successful applications of innate immunotherapy in the clinic have effectively targeted TLRs-induced immune response against cancers [21,52]. TLR2 and TLR4 agonists, Coley toxin (mixture of killed *Streptococcus pyogenes* and *Serratia marcescens* bacteria) and Bacillus Calmette-Guerin have become long-used therapeutic drugs against cancers [53]. Extract of larix leptolepis (ELL), which potentially activates TLR2 and TLR4 signaling, activates bone marrow-derived dendritic cells (BMDCs) to induce tumor-specific cytotoxic T lymphocytes (CTLs) against cancer [54].

Combination therapy with TLR ligands and conventional radiation/chemotherapy is a promising strategy of cancer treatment, which has a much higher growth-inhibitory effect compared with single application and contributes to decrease the clinical dosage of chemotherapeutics [55,56]. Traditional therapy releases tumor antigens, which are subsequently phagocytosed and presented by macrophages and DCs. TLRs stimulation further enhances DC maturation, antigen presentation, and the priming of tumor-specific CTL, which are key issues in effective cancer immunotherapy [57,58,59]. The initiation of adaptive immunity by DCs is also regulated by TLR signaling, in which TLR agonists induce DC maturation, enhance cross-presentation capacity of DCs, and promote robust type I interferon (IFN) production. The TLRs-induced DC maturation further enhances priming of CTL, which depends on the robust type I IFN production [60]. Moreover, type I IFN production and enhanced cross-presentation capacity of DCs activate both innate and adaptive immune system [59]. Therefore, TLRs-regulated DCs bridge innate and adaptive immunity.

## 3. Antitumor Properties of RIG-I-Like Receptors (RLR) Signaling

### 3.1. RIG-I-Like Receptors (RLRs)

**Figure 3:**
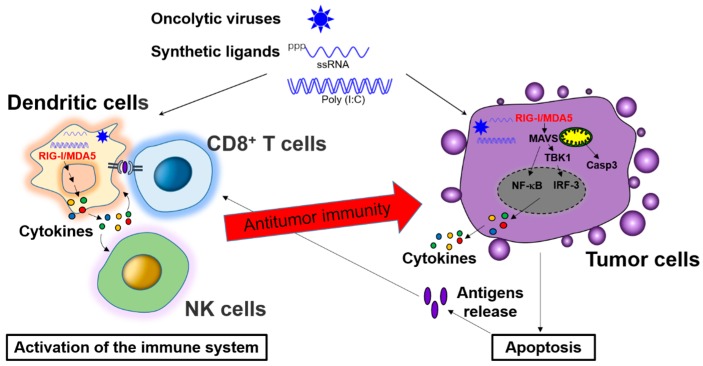
Role of the RIG-1-like Receptors (RLRs) signaling pathway in antitumor immunity. Triggering of RLRs signaling by using oncolytic viruses or synthetic ligands in tumors cells can directly induce tumor cell death via interferon (IFN)-dependent or -independent manner. In addition, these agents are also sensed by host immune cells (primarily dendritic cells), resulting in activation of cluster of differentiation 8^+^ (CD8^+^) T cells or natural killer (NK) cells, which exhibit an antitumor immune response. RLRs-mediated chemokines and cytokines production in the tumor site also contributes to recruitment of effector T cells. Tumor associated antigens from apoptotic tumor cells are presented to CD8^+^ T cell for producing tumor-antigen-specific cytotoxic CD8^+^ T cells.

RIG-I like receptors (RLRs) are a family of DExD/H box RNA helicases that play a major role in pathogenic RNA sensing for initiating antiviral immunity response [61]. So far, three members of the mammalian RLRs family have been identified: RIG-I (retinoic acid inducible gene 1, which is also known as DDX58), MDA5 (melanoma differentiation associated protein 5, which also is named as IFIH1), and LGP2 (laboratory of genetics and physiology 2, which is also known as DHX58), all of these three members are expressed in the cytoplasm of ubiquitous types of cells [62]. These RLRs all share a DexD/H-box RNA helicase domain and a C-terminal domain (CTD), while RIG-I and MDA5, but not LGP2, have an N-terminal caspase activation and recruitment domain (CARD) domain, which is responsible for interacting with a downstream mitochondrial adaptor molecule—MAVS (mitochondrial antiviral signaling protein, which is also named IPS-1) [61]. RIG-I and MDA5 are two major cytosolic receptors for detection of virus-derived RNAs in the cytoplasm. It has been shown that RIG-I binds preferentially to 5′-triphosphorylated RNA (5′-pppRNA or 3pRNA) and short double-strand RNA (dsRNA), while MDA5 recognizes preferentially long dsRNA [62]. In the ligand-free resting state, RIG-I is auto-repressed, the second CARD domain interacts with helicase domain and prevents direct access of any RNA to the helicase domain, and this also hinders the access of ubiquitination enzymes and polyubiquitin binding to the CARDs. Therefore, in the resting state, RIG-I cannot interact with downstream MAVS. Upon virus infection, the viral RNA is recognized by carboxyl-terminal domain (CTD), ATP-dependent conformational change induces a packed complex formation of the helicase domain/CTD with dsRNA, and the CARDs are released from auto-repression. The active RIG-I then interacts with MAVS via RIG-I CARD and MAVS CARD interactions, and promotes MAVS filament formation on the mitochondrial surface. Consequently, MAVS becomes active to stimulate downstream signaling effectors TBK1 (TANK-binding kinase 1) and IKK (inhibitor-κB kinase), which activate transcription factor IRFs (IFN-regulatory factors, mainly IRF-1, IRF-3, and IRF-7) and NF-κB pathway, respectively. Activated IRFs and NF-κB are translocated into the nucleus, and interact with the promoter regions of target genes, including IFNs and inflammatory cytokines [63,64]. MDA5 is activated through a similar mechanism to RIG-I [65]. However, the activation of LGP2 (Laboratory of Genetics and Physiology 2) cannot induce IFNs due to lacking of CARD domain such as RIG-I and MDA5 to interact with MAVS, it is though a regulator in antiviral immune responses. LGP2 has been previously reported to inhibit RIG-I signaling and activity both in vivo and in vitro. In contrast, MDA5-induced signaling transduction is stimulated in the presence of LGP2 [66] (Figure 3).

### 3.2. Activation of RLRs Directly Triggers Cancer Cell Death

Cell death after viral infection is considered to be an important antiviral mechanism that eliminates infected cells and prevents the virus from spreading. Activation of RLRs signaling pathway by using synthetic ligands or virus infection has been shown to induce the production of IFNs, which can induce ISGs (interferon-stimulated genes) for directly producing cell death or modulate apoptosis pathway [67,68,69,70,71,72]. In addition, cell death induction upon activation of RLRs signaling also occurs through IFNs-independent and caspase-3-dependent manner, involving the mitochondrial adaptor molecule-MAVS or transcriptional factor-IRF-3 [73,74,75,76,77,78,79,80,81,82]. Therefore, activation of RLRs can directly trigger cancer cell death and may provide an alternative approach for cancer treatment other than conventional chemotherapy or radiotherapy.

### 3.3. Triggering of RLRs Signaling for Cancer Immunotherapy

In consideration of the fact that immune responses against viruses and tumors share essential features, such as dependence on IFNs and CTLs (cytotoxic T lymphocytes), the idea arises that mimicking a viral infection using RLRs ligands to activate RLRs-signaling mediated immune responses could be exploited for tumor immunotherapy. While RLR ligands are not yet approved by the Food and Drug Administration (FDA) as a treatment for cancers, accumulating evidence strongly suggests that triggering MDA5 or RIG-I would provide a promising approach for cancer immunotherapy.

Because ligands for RIG-I recognition are mostly independent of the RNA sequence and the gene silencing effect is not inhibited by the presence of a 5′-triphosphate (5′ ppp-) moiety in the small interfering RNA molecules (siRNA), several types of 5′ ppp-siRNAs were developed for inducing cell death through activation of the RIG-I signaling pathway and also silencing oncogenic or immunosuppressive targets. It has been reported that systemic administration of 5′ ppp-siRNA for B-cell lymphoma-2 (Bcl-2) silencing, results in melanoma tumor growth inhibition due to the downregulation of Bcl-2 and immune activation of the RIG-I signaling pathway, this anticancer response requires dendritic cells (DC)-dependent production of IFN-α and recruitment of natural killer (NK) cell at the tumor site [28]. The 5′ ppp-siRNA for transforming growth factor beta (TGF-β), which is a cytokine playing a central role in tumor-induced immunosuppression and tumor progression in pancreatic cancer, was also developed for pancreatic cancer therapy. Treatment of mice with established tumors with 5′ ppp-TGF-β siRNA results in reduction of systemic and tumor-associated TGF-β levels, high levels of type I IFN and CXC chemokine ligand 10 (CXCL10) in serum and tumor tissue, systemic immune cell activation and recruitment of activated CD8^+^ T cells to the tumor, profound tumor cell apoptosis, as well as prolonged mice survival [29] (Table 1).

Except for the synthetic RNA ligand for RIG-I activation, it has also been reported that endogenous non-coding RNAs (small nuclear RNAs U1 and U2) translocate to the cytoplasm after ionizing radiation (IR) treatment, thus stimulating the formation of RIG-I: RNA complexes and initiating downstream signaling events trigger IFN-β activity [83]. It is known that the activation of interferon genes by radiation or chemotherapy is associated with a favorable outcome in patients undergoing treatment for cancer. Therefore, the activation of RLR signaling by endogenous RNAs under radiation or chemotherapy may play an important role in antitumor response. The hemagglutinating virus of Japan envelope (HVJ-E) vector derived from inactivated replication-defective Sendai virus also can activate the RIG-I/MAVS signaling pathway by viral RNA fragments and enhance antitumor immunity through activation of effector T cells and NK cells and inhibition of regulatory T cells (Tregs)-mediated immunosuppression [30,31]. Clinical-grade HVJ-E has already been generated and used in clinical trials enrolling melanoma, malignant pleural mesothelioma (MPM), and castration-resistant prostate cancer (CRPC) patients in Japan.

Similarly, triggering MDA5 activation by using synthetic double-stranded RNA-poly(I:C) in ovarian cancer exhibits enhanced expression of HLA-class I, release of cytokines (CXCL10, IL-6, and type I interferon), as well as tumor cell apoptosis. This MDA5-mediated anticancer response requires DC-dependent engulfing of MDA5-activated cancer cells for DC activation and subsequently production of cytokines (CXCL10 and IFN-α) for providing a pro-inflammatory milieu to promote cytolytic activity and IFN-γ secretion of NK cells at the tumor site [32]. Therefore, it has been suggested that activation of RLRs by synthetic or endogenous or virus-derived ligands boosts endogenous NK or CD8^+^ T cell-mediated antitumor immune responses and provides a promising approach for the immunotherapy of cancer (Figure 3).

Comparing with surgery, chemotherapy, or radiation therapy in cancer treatment, there are several benefits to targeting RLRs for cancer immunotherapy. Firstly, activation of RLRs by their specific ligands not only triggers antitumor immunity via IFN-dependent effector T cells and NK cells activation, but also can directly induce apoptosis of tumor cells in an IFN-independent manner. Secondly, it has been indicated that tumor cells are highly sensitive to RLR-induced apoptosis, whereas nonmalignant cells are protected via endogenous Bcl-xL [33,76]. Therefore, triggering RLRs activation by using RLRs ligands may induce tumor cell-specific apoptosis. Furthermore, RLRs activation-mediated apoptosis induction is independent of the p53 mutational status of the tumor cells, which is strongly associated with apoptosis resistance during chemotherapeutic drugs or radiation therapy [84]. Although HVJ-E has been used in clinical trials, targeting RLRs signaling for cancer therapy still has several challenges that need to be addressed before moving into clinical trials, including optimal ligands delivery into the tumor, control of ‘off-target’ effects of 5′ ppp-siRNAs, large-scale ligands production, reducing harmful inflammatory side effects and discovery of effective combination partners, such as chemotherapy, radiation, or immunotherapy with cancer vaccines and checkpoint inhibitors.

## 4. Targeting Stimulator of Interferon Genes (STING) Pathway for Cancer Immunotherapy

### 4.1. STING Signaling Pathway

In addition to sensing of microbial RNAs by RLRs to activate innate immune responses against pathogen infection, the recognition of microbial DNA by DNA sensors is also a major defense mechanism for the detection of a wide variety of pathogens, because all microorganisms contain and need DNA in their life cycle. However, host cells also contain abundant self DNA. To avoid this self DNA recognition, the host has evolved specific mechanisms to distinguish self from non-self DNA based on the location of the DNA or sensors to avoid inappropriate activation of immune responses. After microbial infection, the pathogenic DNA is always presented in cytoplasm, which is different from the nuclear or mitochondrial localization of host DNA, and sensed by the cytosolic DNA sensors to initial innate immune responses. However, under certain pathological conditions, self DNA also can be recognized by innate immune sensors and cause several autoimmune and auto-inflammatory diseases [85].

So far, several cytosolic DNA sensors have been discovered, such as DAI (DNA-dependent activator of IFN-regulatory factors) [86], RNA polymerase III [87], AIM2 (Absent in melanoma 2) [88,89], IFI16 (Interferon-inducible protein 16) [90], DHX9/36 (DEAH (Asp-Glu-Ala-His) box polypeptide 9/36) [91], LRRFIP1 (Leucine-rich repeat flightless-interacting protein 1) [92], DDX41 (DEAD (Asp-Glu-Ala-Asp) box polypeptide 41) [93], Ku70 (70-kDa subunit of Ku autoantigen) [94], DNA-PK (DNA-dependent protein kinase) [95], MRE11 (MRE11 meiotic recombination 11 homolog A) [96], and cGAS (cyclic GMP-AMP Synthase) [97]. After activation by intracellular DNA through direct binding, these DNA sensors trigger the downstream signaling to induce IFNs and other inflammatory cytokine production. Endoplasmic-reticulum (ER)-membrane protein STING (Stimulator of Interferon Genes, also known as MITA, ERIS, MPYS, or TMEM173) is thought to function as an adaptor protein, which links upstream DNA sensors to downstream IRF-3 and NF-κB pathway activation [98,99]. STING also directly senses CDNs (cyclic dinucleotides), including bacterial c-di-GMP (cyclic 3′–5′ diguanylate), c-di-AMP (cyclic 3′–5′ diadenylate) [100,101], 3′,3′-cGAMP (cyclic [G(3′,5′)pA(3′,5′)p]) or cGAS product 2′,3′-cGAMP (cyclic [G(2′,5′)pA(3′,5′)p]) [102,103]. It has been demonstrated that binding with CDNs can induce conformational change and ER to ER-Golgi intermediate compartment/Golgi apparatus trafficking of STING. During this process, the carboxyl terminus of STING recruits TBK1 to phosphorylate IRF-3 and promote IRF-3 dimerization and nuclear translocalization. STING also can activate NF-κB pathway via TRAF6 (TNF receptor-associated factor 6, E3 ubiquitin protein ligase)-TBK1 pathway [104,105]. Finally, the activation of IRF-3 and NF-κB enter into the nucleus and function as transcription factors to induce the expression of IFNs and inflammatory cytokines such as TNF, IL-1β, and IL-6.

### 4.2. Activation of STING Pathway Directly Triggers Cancer Cell Death

As RLRs signaling, activation of STING pathway results in production of IFNs for inducing ISGs to prompt cell death, it also can cause cell death via IFNs-independent manner, which is through STING-mediated IRF-3 interaction with Bcl-2-associated X protein (Bax) on mitochondria to activate the mitochondrial apoptosis pathway dependent on caspases 9 and 3 [106,107,108]. Therefore, triggering of STING signaling can directly induce cancer cell death and may provide a new cancer therapy option. However, it has been demonstrated that apoptotic caspases suppress the activation of STING pathway [109]. Hence, a feedback regulation between STING and apoptosis signaling pathway may exist; trying to release this inhibitory effect of apoptotic caspases on STING may enhance the anticancer activity of STING.

### 4.3. The Role of STING in Cancer Immunotherapy

**Figure 4:**
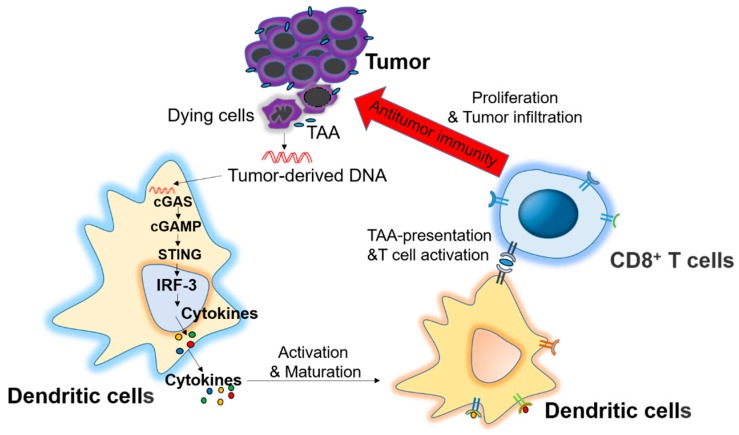
Role of the stimulator of interferon genes (STING) signaling pathway in antitumor immunity. Dying tumor cell-derived DNA is delivered to dendritic cells (DCs) through an unknown mechanism. The DNA is recognized by cytosolic DNA sensor cGAS to produce cGAMP for STING activation and cytokines production, which stimulate the maturation of DCs and stimulate the cross-presentation of tumor associated antigens (TAA) to CD8^+^ T cells, which exhibit an antitumor immunity after proliferation and infiltration into tumor microenvironment.

IFNs play a multifaceted role in the induction of antitumor immunity. In addition to their direct cytotoxic effects on tumor, targeting low doses of type I IFNs to the tumor microenvironment also promotes the maturation and antigen presentation of DCs and boosts endogenous NK or CD8^+^ T cell-mediated antitumor immune responses. Recent studies have demonstrated that STING plays a key role in antitumor immune response [110,111,112]. After transplantation of immunogenic tumors in STING-deficient mice, the tumors grow more rapidly than transplantation in wild-type mice or TRIF-deficient mice. Spontaneous CD8^+^ T cell priming against tumors was also defective in mice lacking STING, but not in those lacking TLRs, MyD88 or MAVS, suggesting cytosolic DNA sensing pathway is involved in controlling tumor growth. It has also been demonstrated that tumor-derived DNA is transferred to host DCs and induces IFNs production through cGAS-STING pathway for CD8^+^ T cell priming [110]. Other studies have also indicated that STING is required for the antitumor effects of radiation or anti-CD47 treatment. The dying tumor-derived DNA after treatment with radiation or antibody to CD47 is delivered to the cytoplasm of DCs to activate the cGAS-STING pathway and induce IFNs production, further bridging the innate and adaptive responses, which is represented by the activation of CD8^+^ T cells [111,112]. Therefore, these studies suggest that activation of STING by tumor-derived DNA for IFNs production and DC-mediated cross-priming is critical for generating adaptive antitumor immunity (Figure 4).

Many kinds of cancers appear to induce a spontaneous adaptive T cell response. However, tumor cells often induce an immunosuppressive microenvironment favoring cancer development. Therefore, targeting of STING pathway by using STING agonists to produce IFNs for enhancing antitumor immune response may provide an alternative strategy for the improvement of cancer immunotherapy. A defined flavonoid compound known as a vascular disrupting agent, 5,6-dimethylxanthenone-4-acetic acid (DMXAA), was shown to have potent antitumor activity in mouse models due to its ability to induce type I IFNs in macrophages [113], the later studies indicated that DMXAA is a direct ligand for mouse STING [114,115]. However, this drug failed to achieve robust antitumor immune responses in humans, detailed analysis revealed that human STING failed to bind to DMXAA due to its polymorphisms, therefore DMXAA-mediated antitumor therapeutic effects in humans was abrogated [114,116]. Although this drug ultimately failed in humans, studies in mouse models suggested that DMXAA treatment can effectively prime CD8^+^ T cell response and enhance antitumor immunity [117,118]. In addition, delivery of cyclic dinucleotides (such as cGAMP, c-di-GMP, and c-di-AMP) into tumor-bearing mice has been shown to substantially inhibit tumor growth and improves the survival of the mice due to STING-mediated boosting of antitumor immune responses [34,35,110,119,120,121].

These results indicate that STING activation by cGAMP and potentially other ligands may be an attractive treatment for antitumor immunotherapy. One of the STING ligands-MIW815, is now in phase I clinical trials. However, there are still several challenges yet to be fully addressed, because several studies found a risk of STING activation in cancer immunotherapy. It has been reported that activation of the cGAS-STING pathway can lead to tolerogenic responses and promote the growth of tumors with low antigenicity through the induction of indolamine 2,3-dioxygenase (IDO) [122,123]. A recent study has also indicated that brain metastatic cancer cells use carcinoma–astrocyte gap junctions to transfer cGAMP to neighboring astrocytes, activating the STING pathway and producing inflammatory cytokines, which support tumor growth and chemoresistance [124]. These studies give us a warning that activation of STING signaling may play a dual role in controlling antitumor and pro-tumor immunity. Fortunately, an optimal combination of STING agonists with other treatments, including irradiation, chemotherapy, or blockade of immune-system checkpoints may achieve a good clinical outcome. Recent studies have indicated that the combination of cGAMP or other CDNs with irradiation, chemotherapy, or immune-system-checkpoint inhibitors (such as antibodies to cytotoxic T lymphocyte antigen 4 (CTLA4), programed cell death-1 (PD-1) and programmed death-ligand 1 (PD-L1) produces synergistic antitumor effects [34,36,111].

## 5. Combined Cancer Immunotherapies with Synergistic Activation of Innate and Adaptive Immune Responses

Many cancer immunotherapeutic strategies enhancing distinct aspects of the immune system are showing clinical promise. Meanwhile, powerful synergistic effects in cancer immunotherapy have been evidenced when innate and adaptive immunity are combined in use, indicating that the engagement of both innate and adaptive immune responses is necessary for efficient cancer immunotherapy.

### 5.1. Activation of Adaptive Immune Responses by Innate Immunity in Cancer Therapy

The current barrier to effective cancer treatments is that cancer itself is highly immunosuppressive to both innate and adaptive responses [36]. Although adaptive immunity is deemed to play a critical role in the control of tumors, innate immune cells, especially macrophages and NK cells, are much less suppressed [52,125]. Moreover, triggering of innate immunity is able to induce inflammatory cascade pathways to activate ineffective adaptive immune elements, in which induction of innate immunity acts as a bridge to adaptive immunity [52]. Therefore, targeting innate immunity is an attractive immunotherapeutic strategy.

Initiation of innate immune responses can lead to subsequent long-term adaptive immunity against cancer. Thus, integration of innate immune activation strategies into combination therapies for cancer treatment will lead to more effective and long term clinical benefit. Evidence has demonstrated that manipulating tumor-associated macrophages (TAM) or myeloid derived suppressor cells (MDSC) to break the systemic and local tumor-induced immunosuppression potentially support cytotoxic T-cells (CTL)-mediated cancer immunotherapy [126,127].

Monotherapy for most tumor patients is not sufficient for complete cancer eradication, perhaps due to compensatory immune evasion pathways. Negative regulatory receptor programed cell death-1 (PD-1) is an inhibitory molecule that is expressed on the surface of activated immune cells (e.g., CD8^+^ T cells) and binds to its ligand PD-L1, which is expressed on multiple malignant cells [128,129]. Trials of PD-1 and PD-L1 inhibitors have shown objective and durable responses in advanced solid tumors [130,131]. Notably, tumor regression following therapeutic PD-1/PD-L1 blockade requires adequate antigenic T-cell response, tumor-infiltrating lymphocytes, and cytokine secretion such as IFN [132], which reflects the cooperation between innate and adaptive immunity. So future trials may consider a combination of PD-1/PD-L1 inhibitors with innate and adaptive immune activators.

### 5.2. Enhancement of Innate Immune Effectors Activation by Adaptive Immunity

Common γ chain cytokines, namely IL-2, IL-4, IL-7, IL-9, IL-15, and IL-21, produced by activated immune cells influence the activation of innate immune effector cells [133]. IL-2 is a cytokine with a wide range of biological activity mainly secreted by activated CD4^+^ T cells. Exposure of peripheral blood mononuclear cells (PBMCs) to IL-2 could generate lymphokine-activated killer (LAK) cells that have the ability of spontaneous lysis of tumor cells. The IL-2-induced LAK activity has been successfully applied in immunotherapy against melanoma and renal cell carcinoma [134]. IL-15 has been reported to be another common γ chain cytokine that could be safely administered to patients with metastatic malignancy. The IL-15 administration significantly promotes NK cell activation in cancer patients [135].

Antibody-based targeted therapy is one of the safest immunotherapeutics for cancer patients. Clinical studies have demonstrated that effective tumor-specific monoclonal antibodies (mAbs) induce direct tumor cell death involving innate immune pathways; some tumor-reactive mAbs function by the aid of complement dependent cytotoxicity, while most mAbs with antitumor efficacy involve innate effector cells via antibody dependent cell-mediated cytotoxicity (ADCC) [52]. Thus, when mAbs are combined with innate immune-stimulation, clinical benefits can be significantly augmented.

The classically activated (M1) macrophages play a tumor-destroying role and the M1 macrophage densities in the tumor islets are highly associated with the patient’s survival [136,137]. Anti-CD40 agonist mAb combined with TLR activation has been shown to induce augmented function of M1 macrophage and promote antitumor effects [57,138]. In addition, certain chemotherapies can synergize with anti-CD40 and TLR agonists to change tumor-associated macrophages from pro-tumor M2 phenotype to anti-tumor M1 phenotype, resulting in anti-tumor effects in murine melanoma and neuroblastoma models [139].

## 6. Conclusions

The innate immune signaling pathways play a key role in tumor escape from immune surveillance, and thus triggering the innate immune pathways will likely be critical in strategies of cancer immunotherapy. Accumulating evidence has indicated that targeting certain innate immune signaling pathways, especially TLRs, RLRs, and STING signaling pathways, is a promising cancer immunotherapeutic approach. Therefore, further insight into the mechanisms of TLRs, RLRs, and STING-mediated innate immune signaling in cancer immune evasion, tumorigenesis, and cancer development may lead to discovery of novel therapeutic targets for cancer therapy. Besides this, considering the compensatory immune mechanism, together with integration of the strategies targeting both innate and adaptive immune systems for cancer treatment will contribute to more powerful synergistic effects and long term clinical benefits.

## Figures and Tables

**Table 1:** Overview of promising agents that trigger the Toll-like receptors (TLR), RIG-I-like receptors (RLR) and stimulator of interferon gene (STING) pathway for cancer immunotherapy.

Promising Agents	Receptors	Cancer Types	References
Bacillus Calmette-Guérin (BCG)	TLR2/4	Bladder cancer	[19]
monophosphoryl lipid A(MPL)	TLR4	Cervical cancer	[22]
Imiquimod	TLR7	Breast cancer	[23]
Flagellin-derived CBLB502 (Entolimod)	TLR5	Hepatoma	[24]
852A	TLR7	Hematologic malignancy	[25]
CpG ODN	TLR9	Glioblastoma	[26]
poly(I:C)/poly-ICLC	TLR3	Multiple cancer types	[27]
5′ ppp-siRNA for Bcl-2	RIG-I	Melanoma	[28]
5′ ppp-siRNA for TGF-β	RIG-I	Pancreatic cancer	[29]
HVJ-E	RIG-I	Prostate cancer, gliomas	[30,31]
poly(I:C)	MDA5	Ovarian cancer, Pancreatic cancer	[32,33]
cGAMP	STING	Colon cancer	[34]
c-di-GMP	STING	Melanoma	[35]
STINGVAX	STING	Melanoma	[36]

poly(I:C): polyinosinic-polycytidylic acid; CpG ODN: CpG-containing oligodeoxynucleotides; Bcl-2: B-cell lymphoma-2; TGF-β: transforming growth factor beta; HVJ-E: hemagglutinating virus of Japan envelope; cGAMP: cyclic AMP-GMP; RIG-I: retinoic acid-inducible gene-I; siRNA: small interfering RNA.
